# Organocatalytic Packed-Bed Reactors for the Enantioselective Flow Synthesis of Quaternary Isotetronic Acids by Direct Aldol Reactions of Pyruvates

**DOI:** 10.3390/molecules30020296

**Published:** 2025-01-13

**Authors:** Lorenzo Poletti, Carmela De Risi, Daniele Ragno, Graziano Di Carmine, Riccardo Tassoni, Alessandro Massi, Paolo Dambruoso

**Affiliations:** 1 Department of Environmental and Prevention Sciences, University of Ferrara, Via L. Borsari, 46, 44121 Ferrara, Italy; lorenzo.poletti@unife.it (L.P.); graziano.dicarmine@unife.it (G.D.C.); 2 Department of Chemical, Pharmaceutical and Agricultural Sciences, University of Ferrara, Via L. Borsari, 46, 44121 Ferrara, Italy; carmela.derisi@unife.it (C.D.R.); daniele.ragno@unife.it (D.R.); 3 Institute for Organic Synthesis and Photoreactivity of the Italian National Research Council, Area della Ricerca di Bologna, Via P. Gobetti, 101, 40129 Bologna, Italy; riccardo.tassoni@isof.cnr.it

**Keywords:** flow chemistry, organocatalysis, aldol reaction, butenolides, pyruvates

## Abstract

The utilization of the homogeneous (*S*)-2-pyrrolidine-tetrazole organocatalyst (Ley catalyst) in the self-condensation of ethyl pyruvate and cross-aldol reactions of ethyl pyruvate donor with non-enolizable pyruvate acceptors, namely the sterically hindered ethyl 3-methyl-2-oxobutyrate or the highly electrophilic methyl 3,3,3-trifluoropyruvate, is described as the key enantioselective step toward the synthesis of the corresponding biologically relevant isotetronic acids featuring a quaternary carbon functionalized with ester and alkyl groups. The transition from homogeneous to heterogeneous flow conditions is also investigated, detailing the fabrication and operation of packed-bed reactors filled with a silica-supported version of the pyrrolidine-tetrazole catalyst (SBA-15 as the matrix).

## 1. Introduction

**Figure 1:**
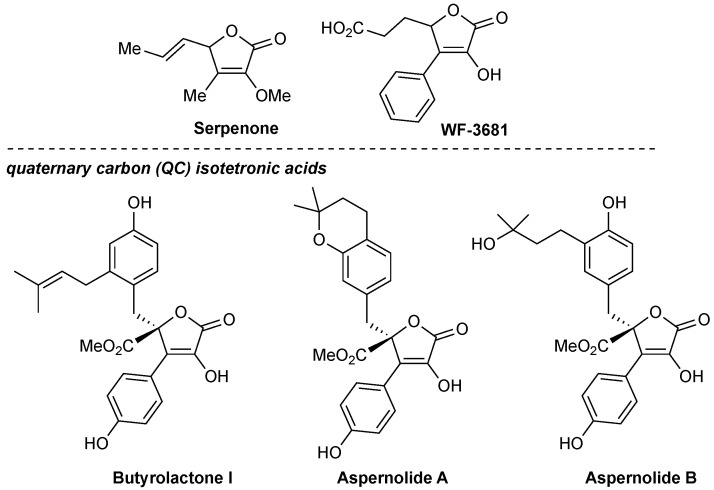
Representative bioactive isotetronic acids, including natural QC-isotetronic acids.

**Scheme 1:**
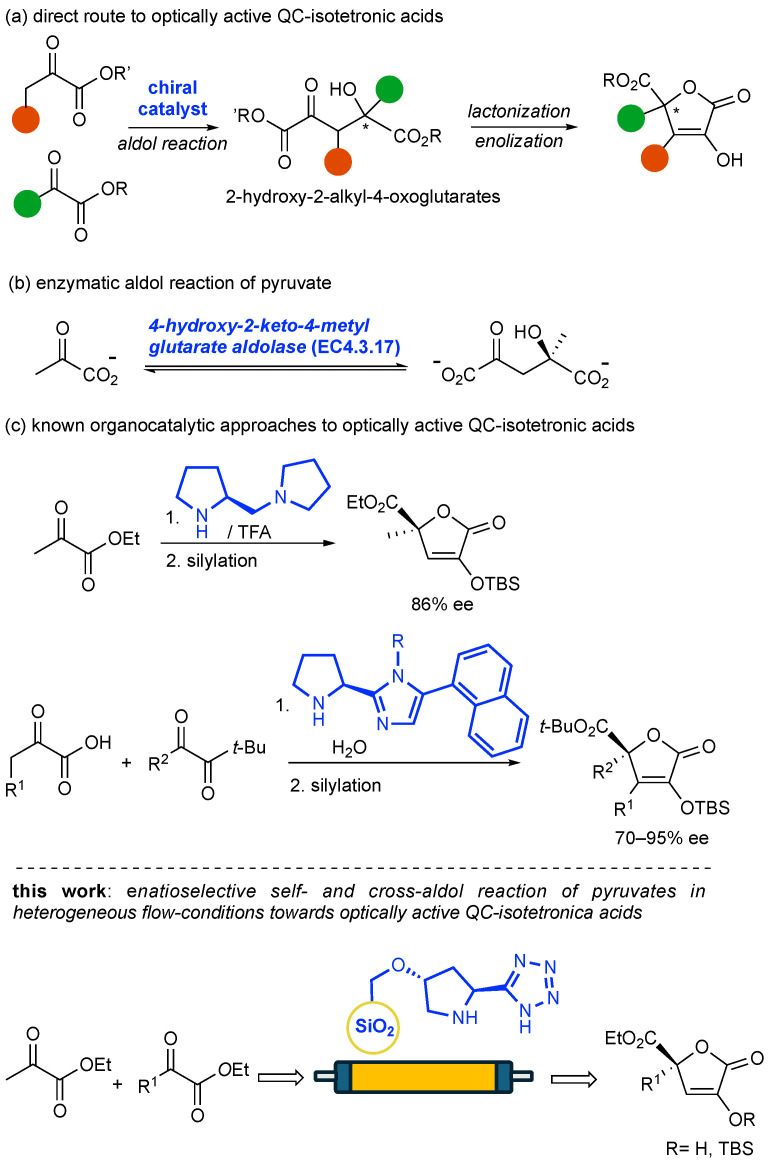
Enzymatic and organocatalytic strategies towards optically active QC-isotetronic acids [9,10].

Isotetronic acids are classified as butenolides and are significant for their synthetic applications and inherent biological properties [1]. Serpenone has been recognized as a metabolite of the higher fungus Hypoxylon serpens and has demonstrated efficacy in the treatment of diabetic neuropathy [2]. WF-3681, an additional fungal metabolite extracted from Chaetomella raphigera, has been reported to possess aldose reductase inhibitory action (Figure 1) [3]. A particular class of optically active isotetronic acids displaying quaternary carbon (QC) functionalized with an ester group and an alkyl substituent (QC-isotetronic acids) has been shown to have exceptional bioactivity. This is the case of butyrolactone I and Aspernolides A/B, natural products isolated from the fungi Aspergillus terreus, which exhibit very promising antitumor activity against colon, lung, prostatic, and pancreatic carcinoma (Figure 1) [4,5,6]. Consequently, the development of an efficient and stereoselective method to access this bioactive scaffold would be highly desirable for new lead identification and optimization. The enantioselective catalytic self- and cross-aldol reactions of pyruvates leading to chiral quaternary α-hydroxy esters (2-hydroxy-2-alkyl-4-oxoglutarates), followed by a lactonization/enolization sequence, certainly represent the most straightforward route to explore the (stereo)chemical diversity of optically active QC-isotetronic acids (Scheme 1a). The natural enzyme 4-hydroxy-2-oxo-4-methyl-glutarate aldolase (EC4.1.3.17) effectively promotes the self-condensation of pyruvate with complete stereocontrol in the biosynthesis of the corresponding α-hydroxy acid (Scheme 1b) [7]. To the best of our knowledge, however, enzymatic aldol reactions based on different α-keto ester/acid combinations have not yet been documented in the literature, thus demonstrating how enzyme substrate specificity may represent a limitation of biocatalysis on some occasions. Indeed, the first asymmetric homo- and cross-aldol condensations of α-keto esters leading to protected QC-isotetronic acids (O-*t*-butyldimethylsilyl derivatives) were described by the group of Jørgensen using a chiral copper(II)/bisoxazoline metal catalyst [6,8]. Our group also contributed to this area of research, describing in 2004 the first organocatalytic approach to the enantioselective self-aldol reaction of ethyl pyruvate promoted by a proline-like catalyst; by this strategy, the corresponding isotetronic acid was obtained in good yield and enantioselectivity (Scheme 1c) [9]. More recently, Liu, Li, and their co-workers showed the ability of water in the presence of bulky proline-imidazole amphiphilic organocatalysts to differentiate the nucleophilicity between α-keto esters and α-keto acids and thus obtain a range of protected, optically active QC-isotetronic acids (Scheme 1c) [10]. Today, organocatalysis in heterogeneous conditions has proven to be a robust methodology for highly selective, metal-free syntheses of biologically relevant molecules and active pharmaceutical ingredients (APIs), with advantages compared to traditional solution-phase approaches in terms of product isolation, catalyst recycling, and process intensification using continuous flow reactors [11]. Accordingly, following our interest in the application of flow chemistry as an enabling technology for the efficient and sustainable production of valuable (bio)molecules [12,13], we report here on model self- and cross-aldol flow reactions of pyruvates by operating packed-bed reactors functionalized with the silica-supported 5-(pyrrolidin-2-yl)tetrazole organocatalyst, which is a ‘privileged’ catalytic structure deeply investigated by our group in its heterogenous versions [12,14,15]. In this contribution, we also outline the possible application of a set of polymer-supported reagents and sequestrants to facilitate the isolation of QC-isotetronic acids in their biologically active hydroxy-free form.

## 2. Results and Discussion

In our previous study [9], we found that the self-aldol reaction of ethyl pyruvate 1 occurs effectively under the action of a diamine-protic acid catalyst, namely the (S)-(+)-1-(2-pyrrolidinylmethyl)pyrrolidine/trifluoroacetic acid (TFA) couple **3**, in i-PrOH at room temperature. Hence, the behavior of **1** in the presence of other highly active organocatalysts operating via either HOMO or LUMO activation mechanisms [16] was examined to gather further data on this transformation in the solution phase before moving to heterogeneous conditions. Accordingly, the Arvidsson–Ley–Yamamoto (S)-2-pyrrolidine-tetrazole **4** (often simply referred to as Ley catalyst) [17,18], and the MacMillan imidazolidinone **5** [19] were considered, and their activity evaluated in different solvents (Table 1) after conversion of the crude aldol reaction mixture into the protected O-TBS isotetronic acid **2** (TBSCl, Imidazole, DCM). Indeed, it is important to emphasize that the purification by standard procedures (aqueous workup and chromatography) of unprotected QC-isotetronic acids in real isolated yields is reported to be precluded due to the lability of this class of molecules under aqueous and acid conditions [1,9,10]. Catalyst **4** proved to be less effective than **3**, as the best yield (46%) and ee (62%) values of **2** (entry 4) were obtained in i-PrOH and with a 10 mol% optimized loading of the catalyst. On the other hand, imidazolidinone **5** (30 mol%) in the presence of 1 equiv. of TFA or AcOH in different solvents (i-PrOH, DMSO, MeCN) did not promote the homoaldolization of **1** at all.

To create a model cross-aldol reaction of pyruvates that was selectively controlled by steric effects, we then proceeded with our propaedeutic solution phase study by identifying a suitable α-keto ester that could serve as the electrophilic coupling partner of ethyl pyruvate **1**. After a brief screening of substituted α-keto esters (Supplementary Materials), this reagent, which needed to be scarcely or not at all enolizable, was identified in the ethyl 3-methyl-2-oxobutyrate **6** (Table 2). Therefore, the target aldol reaction of **1** with **6** was studied in i-PrOH at room temperature by changing the molar ratio of the reactants, the catalyst, and the loading of the latter. The reaction outcome was monitored through the formation of the chiral O-silyl isotetronic acids **2** and **7**, as detected by NMR analysis. A first indication of the occurrence of the cross-aldol reaction was obtained by mixing the two α-keto esters in 1:1 molar ratio in the presence of the diamine/TFA couple **3** or the pyrrolidine-tetrazole **4** with a 30 mol% loading of each catalyst (Table 2, entries 1 and 2). However, under these conditions, the homo-aldol reaction of **1** still prevailed, as shown by the higher yields of isotetronic acid **2** with respect to **7**. This indicates that the intrinsic acceptor ability of ethyl pyruvate **1** is superior to that of the alkyl branched α-keto ester 6, as could be expected based on steric considerations. Hence, attempts to affect the kinetics of the system were carried out by employing an excess of **6** with respect to **1** and by slowly adding the latter to the solution of the former and the catalyst using a syringe pump. Better results were obtained with catalyst **4** than with **3**, whereas **5**/TFA was again ineffective (entry 7). In fact, with a 30 mol% loading of **4**, the QC-isotetronic acid **7** was isolated in very high enantiomeric purity (98% ee) and in good yet superior yield (40%) to that of **2** (34%; entry 4). A lower loading (20 mol%) of catalyst **4** resulted in a lower yield and optical purity of both products **2** and **7** (entry 5). As detected in the control experiments, the ratio of **2** and **7** was the same at a shorter reaction time, i.e., **3** instead of **7** days (entry 6), thus indicating that the self- and cross-aldol intermediates were stable under the conditions employed.

**Scheme 2:**
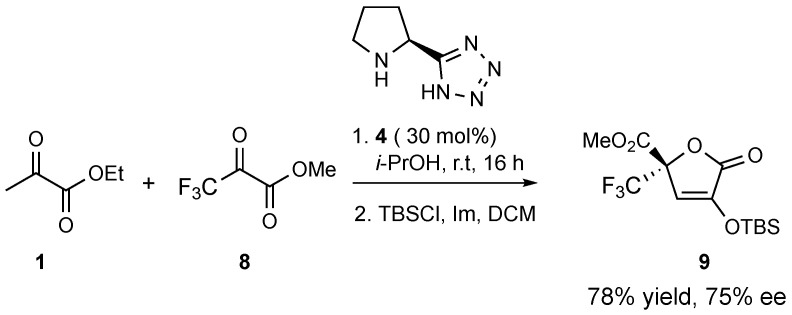
Synthesis of protected isotetronic acid **9** by cross-aldol reaction of ethyl pyruvate **1** and methyl trifluoropyruvate **8** promoted by organocatalyst **4**.

The optimal conditions for entry 4 (30 mol% of **4**, i-PrOH, r.t.) were then applied to the cross-aldol reaction of ethyl pyruvate **1** with the non-enolizable and highly electrophilic methyl 3,3,3-trifluoropyruvate **8** (Scheme 2). The selectivity of this model coupling was controlled by favorable electronic effects; specifically, the protected isotetronic acid **9** was obtained in good yield (78%) and ee (75%) in a shorter reaction time (16 h) with only 2 equivalents of acceptor **8**, while the formation of a minor amount of **2** (14%) was also detected. Notably, the registered enantioselectivity was much higher than that recently observed for the same cross-aldol process catalyzed by a primary amine-derived catalyst (48% ee) [20].

As far as the stereoselectivity of the aldol processes is concerned, the absolute configuration of isotetronic acid **2** was determined to be (S) by comparison of the optical rotation value with that previously reported for the same compound [6,8], while that of isotetronic acids **7** and **9** was assigned by analogy and a common mechanistic hypothesis for the key aldol step (vide infra).

**Scheme 3:**
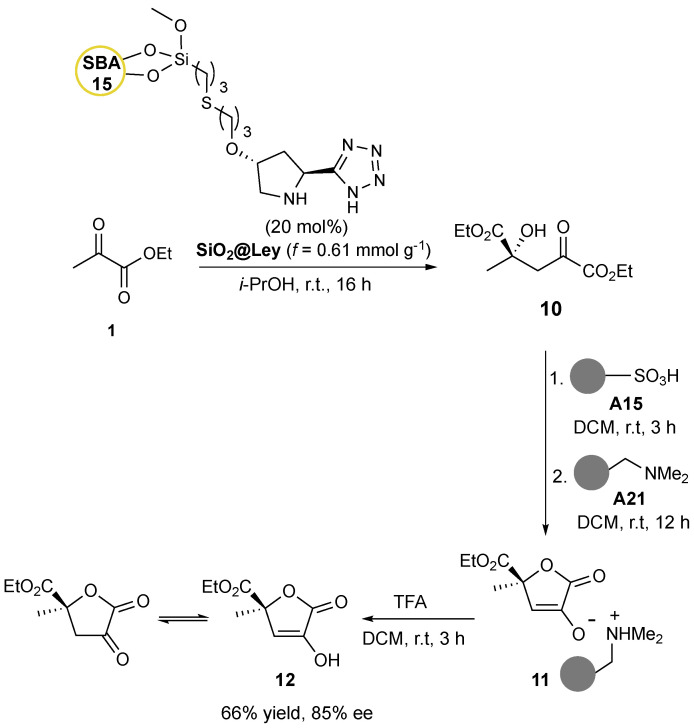
Synthesis of the hydroxy-free QC-isotetronic acid **12**.

With the above data in hand and the goal of improving the efficiency of QC-isotetronic acid production, the best-performing tetrazole organocatalyst **4** was supported on SBA-15 silica material to give the heterogeneous SiO_2_@Ley catalyst following the procedure previously described by our group (loading f = 0.61 mmol g^−1^; median pore size = 5.36 nm) [12,14]. After a brief screening of different polarity solvents aimed at maximizing conversion efficiency and enantioselectivity (i-PrOH, MeCN, DMSO, DCM, Et_2_O, toluene), the self-aldol of ethyl pyruvate **1** was optimized in i-PrOH with 20 mol% of SiO_2_@Ley, leading to the 2-hydroxy-2-alkyl-4-oxoglutarate **10** in 76% yield and almost pure form (^1^H NMR analysis) after catalyst filtration and evaporation of unreacted **1** (Scheme 3). At this stage, our approach to the biologically active hydroxy-free isotetronic acid **12** was attempted by applying the so-called catch-and-release technique [21]. Accordingly, the self-aldol mixture was first treated with the acidic resin A15 to promote the lactonization of the functionalized glutarate **10** to the isotetronic acid **12**, which in turn was sequestered by the basic resin A21 as the corresponding ammonium salt **11**. After filtration of soluble by-products, the solid material was treated with TFA to release the QC-isotetronic acid **12** in equilibrium with its dicarbonyl form in 66% overall yield from **1** and 85% ee. Notably, the SiO_2_@Ley catalyst outperformed its homogeneous counterpart **4** in terms of enantioselectivity (85% vs. 62% ee), suggesting a matrix effect on transition state geometry, possibly due to favorable hydrogen bonding interactions. An in-depth examination of the impact of the silica support on the stereochemical outcome of aldol reactions of pyruvates promoted by the pyrrolidine-tetrazole unit is currently underway in our laboratories. Unfortunately, the direct lactonization in basic conditions with A21 mainly resulted in the enolization of **10** and the formation of a complex reaction mixture that could not be elaborated into the target butenolide **12**.

**Figure 2:**
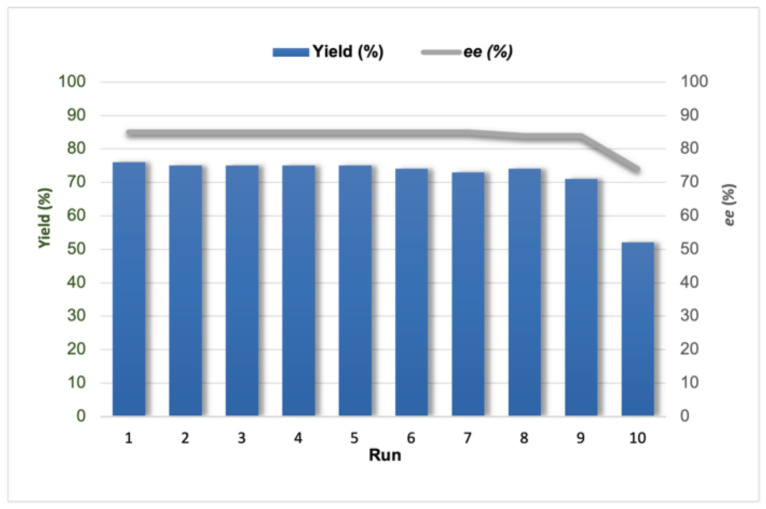
Recycling experiments (synthesis of **10** by homocoupling of **1** with 20 mol% of SiO_2_@Ley).

Additionally, the recyclability of the SiO_2_@Ley catalyst was also investigated (Figure 2), and it was observed that the conversion efficiency remained constant, with only a small drop in enantioselectivity after the tenth run of the aldol reaction.

The asymmetric self- and cross-aldol reactions of pyruvates promoted by heterogeneous (organo)catalysts in a continuous-flow regime is a completely unexplored research domain, potentially providing novel avenues for the intensification of isotetronic acid synthesis. Therefore, based on our experience with the operation of organocatalytic fixed-bed reactors [12,14,15], we next investigated the transition of the **1** homocoupling from batch to continuous-flow conditions. Initially, the mesoreactor R was fabricated by slurry packing the SiO_2_@Ley catalyst (i-PrOH as solvent) into a stainless steel column (10 cm in length, with an internal diameter of 2.1 mm). Then, the dead volume (V_0_) and the total porosity (ε_tot_) of the reactor were determined using pycnometry to calculate the residence time at different flow rates (Table 3).

**Figure 3:**
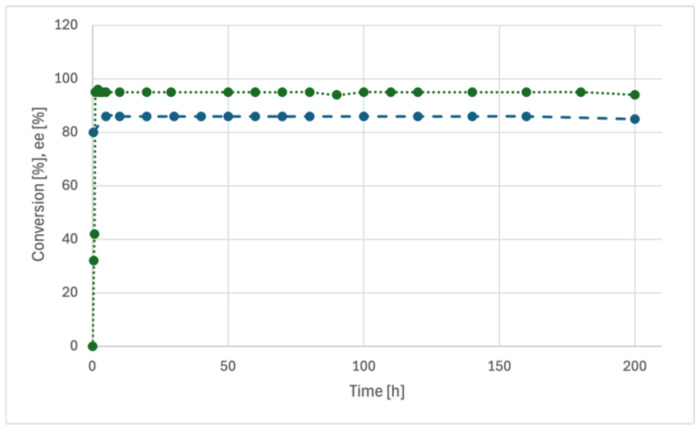
Long-term stability experiment (homocoupling of **1**). Conversion [%], dotted line; ee [%], dashed line.

The first goal of the flow studies was to completely convert the ethyl pyruvate **1** in a single pass-flow mode to facilitate the post-reaction phase and the subsequent elaboration of the crude aldol mixture into the protected isotetronic acid **2** (Table 4). After some experimentation (only selected data are reported), full conversion was achieved by pumping a 0.14 M solution of **1** with a flow rate of 5 μL min^−1^ (residence time = 49 min). Under these conditions (entry 3), the steady-state regime was reached in approximately one hour and a turnover frequency (TOF) of 4.8 mmol_prod_ d^−1^ mmol_cat_^−1^ was recorded. Importantly, the mesoreactor **R** could be operated for ca. 200 h with unaltered values of conversion efficiency (>95%) and enantioselectivity (85% ee; Figure 3), thereby confirming the already noted long-term stability of the catalytic SiO_2_@Ley packing material in a flow regime [12,14]. Gratifyingly, by suitably adjusting the initial reagent concentrations (entries 4–5), the selected acceptors **6** and **8** effectively reacted with the donor **1**, achieving complete instant conversions at a flow rate of 5 μL min^−1^. In the **1**/**6** coupling, the excess of unreacted acceptor **6** was conveniently recovered by fractional distillation at reduced pressure for minimal waste generation. Significantly, the SiO_2_@Ley catalyst reproduced the level of stereoselectivity imparted by the homogeneous **4**, yielding the target isotetronic acids **7** and **9** in 98% and 75% ee, respectively.

**Scheme 4:**
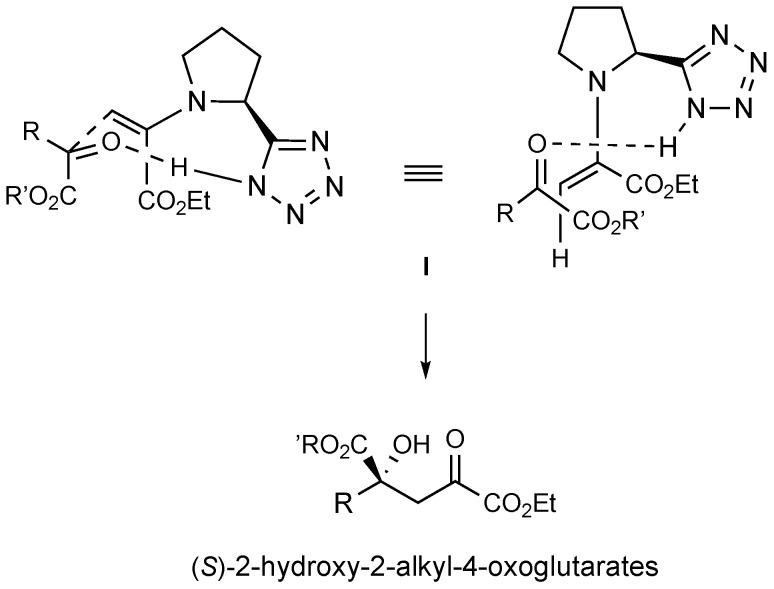
Proposed mechanistic model.

To account for the formation of S-configured isotetronic acids **2**, **7**, and **9** in the aldol reaction of pyruvates under pyrrolidine-tetrazole catalysis, we propose the chairlike transition state (TS) **I**, in analogy with standard proline-catalyzed transformations (Scheme 4) [22,23,24]. Accordingly, the (*S*)-2-pyrrolidine-tetrazole moiety is expected to direct a nucleophilic Si-facial attack of the α-keto ester acceptor by the enamine intermediate generated in situ from the pyruvate donor.

## 3. Materials and Methods

Commercially available reagents were purchased from commercial sources and used without any subsequent purification. The solvents used for starting preparations were distilled from appropriate drying agents and stored over 3 Å molecular sieves. ^1^H, ^13^C, and ^19^F NMR spectra were recorded on Varian Mercury Plus 400 (Varian Inc., Palo Alto, CA, USA) and Brucker Magnet System Ascend 500 MHz (Bruker, Billerica, MA, USA) spectrometers in CDCl_3_ at room temperature. ^13^C{^1^H} NMR spectra were recorded in ^1^H broad-band decoupled mode, and chemical shifts (δ) are reported in parts per million (ppm) relative to the residual solvent peak. Reactions were monitored by TLC on silica gel 60 F254. Flash column chromatography was performed on silica gel 60 (230−400 mesh). High-resolution mass spectra (HRMS) were recorded in positive ion mode by an Agilent 6520 HPLC-Chip Q/TF-MS nanospray instrument (Agilent Technologies, Santa Clara, CA, USA) using a time-of-flight, quadrupole, or hexapole unit to produce spectra. Enantiomeric excesses were measured using HPLC DAD/RID, Agilent Technologies, series 1260 Infiniti II (Agilent Technologies, Santa Clara, CA, USA). Flow reactions were performed using an HPLC pump (Agilent Technologies, series 1100, Santa Clara, CA, USA) and syringe pump (Harvard Apparatus, syringe pump model 22, Harvard Apparatus, Holliston, MA, USA).

### 3.1. Self-Aldol Reaction of Ethyl Pyruvate 1 Promoted by the Ley Catalyst 4 (Table 1, Entry 4)

Ethyl (*S*)-4-((tert-butyldimethylsilyl)oxy)-2-methyl-5-oxo-2,5-dihydrofuran-2-carboxylate (**2**). To a stirred solution of organocatalyst **4** (12 mg, 0.09 mmol) in *i*-PrOH (1 mL), ethyl pyruvate **1** (100 μL, 0.90 mmol) was added dropwise. The mixture was stirred at room temperature for 18 h and then concentrated. The resulting residue was dissolved in anhydrous CH_2_Cl_2_ (4 mL), and then imidazole (184 mg, 2.70 mmol) and tert-butyldimethylsilyl chloride (271 mg, 1.80 mmol) were added sequentially. The mixture was stirred at room temperature for 18 h, and then concentrated and eluted from a column of silica gel with 6:1 cyclohexane-Et_2_O to give **2** (124 mg, 46%; ee = 62%) as a clear colorless oil. ^1^H NMR (400 MHz, CDCl_3_) δ = 6.22 (s, 1H), 4.21 (q, *J* = 7.1 Hz, 2H), 1.68 (s, 3H), 1.27 (t, *J* = 7.1 Hz, 3H), 0.95 (s, 9H), 0.24 (s, 6H). ^13^C{^1^H} NMR (101 MHz, CDCl_3_) δ = 169.0, 168.1, 143.2, 124.4, 81.6, 62.4, 25.4, 22.9, 18.3, 14.0, −4.8. Spectroscopic data are in accordance with the literature [6,8,9].

### 3.2. Cross-Aldol Reaction of Ethyl Pyruvate 1 with Ethyl 3-Methyl-2-Oxobutyrate 6 Promoted by the Ley Catalyst 4 (Table 2, Entry 4)

Ethyl (S)-4-((tert-butyldimethylsilyl)oxy)-2-isopropyl-5-oxo-2,5-dihydrofuran-2-carboxylate (**7**). To a stirred solution of the organocatalyst **4** (21 mg, 0.15 mmol) in *i*-PrOH (1 mL), ethyl 3-methyl-2-oxobutyrate **6** (655 μL, 4.50 mmol) was added in one portion. To the resulting mixture, a solution of ethyl pyruvate **1** (50 μL, 0.45 mmol) in *i*-PrOH (2 mL), was added over 36 h by means of a syringe pump. The mixture was stirred at room temperature for an additional 132 h and then concentrated. The resulting residue was dissolved in anhydrous CH_2_Cl_2_ (4 mL), and then imidazole (92 mg, 1.35 mmol) and tert-butyldimethylsilyl chloride (136 mg, 0.90 mmol) were added sequentially. The mixture was stirred at room temperature for 18 h, and then concentrated and eluted from a column of silica gel with 9:1 cyclohexane-AcOEt to give **7** (59 mg, 40%; ee = 98%) as a clear colorless oil. [α]_D_^20^ = –48.5 (c 0.8, CHCl_3_). HPLC analysis Chiralpak^®^ IA (*n*-Hexane:*i*-PrOH 97:3, flow rate 1 mL min^−1^, 242 nm, 20 °C) t_1_ = 4.2 min (minor), t_2_ = 4.4 min (major), 99.08:0.92 er. ^1^H NMR (400 MHz, CDCl_3_) δ = 6.16 (s, 1H), 4.30–4.18 (m, 2H), 2.45 (hept, *J* = 6.9 Hz, 1H), 1.29 (t, *J* = 6.9 Hz, 3H), 1.02 (d, *J* = 6.9 Hz, 3H), 0.96 (s, 9H), 0.88 (d, *J* = 6.9 Hz, 3H), 0.25 (s, 1H), 0.24 (s, 1H). ^13^C{^1^H} NMR (101 MHz, CDCl_3_): δ = 169.0, 168.3, 143.2, 123.1, 87.4, 62.1, 33.7, 25.4, 18.3, 17.0, 15.7, 14.1, −4.9. HRMS (ESI) m/z: [M + H]^+^ calcd. for C_16_H_29_O_5_Si^+^ 329.4880, found 329.4801.

### 3.3. Cross-Aldol Reaction of Ethyl Pyruvate 1 with Methyl Trifluoropyruvate 8 Promoted by the Ley Catalyst 4 (Scheme 2)

Methyl (*S*)-4-((tert-butyldimethylsilyl)oxy)-5-oxo-2-(trifluoromethyl)-2,5-dihydrofuran-2-carboxylate (**9**). To a stirred solution of organocatalyst **4** (21 mg, 0.15 mmol) and methyl 3,3,3-trifluoropyruvate **8** (92 μL, 0.90 mmol) in *i*-PrOH (2 mL), ethyl pyruvate **1** (50 μL, 0.45 mmol) was added dropwise. The mixture was stirred at room temperature for 16 h and then concentrated. The resulting residue was dissolved in anhydrous CH_2_Cl_2_ (4 mL), and then imidazole (184 mg, 2.70 mmol) and tert-butyldimethylsilyl chloride (271 mg, 1.80 mmol) were added sequentially. The mixture was stirred at room temperature for 18 h, and then concentrated and eluted from a column of silica gel with 6:4 pentane-toluene to give **9** (119 mg, 78%; ee = 75%) as a clear colorless oil. [α]_D_^20^ = +9.6 (c 1.2, CHCl_3_). HPLC analysis Chiralpak^®^ IA (*n*-Hexane:*i*-PrOH 98:2, flow rate 1 mL min^−1^, 242 nm, 20 °C) t_1_ = 10.8 min (major), t_2_ = 11.3 min (minor), 87.51:12.49 er. ^1^H NMR (400 MHz, CDCl_3_) δ = 5.81 (s, 1H), 3.80 (s, 3H), 0.90 (s, 9H), 0.18 (s, 6H). ^13^C{^1^H} NMR (126 MHz, CDCl_3_) δ = 166.4, 163.9, 144.0, 123.3 (q, *J* = 286.8 Hz), 111.9, 94.4 (q, *J* = 33.3 Hz), 61.9, 26.4, 14.1, −3.0. ^19^F NMR (471 MHz, CDCl_3_) δ = −77.71. HRMS (ESI) m/z: [M + H]^+^ calcd. for C_13_H_20_F_3_O_5_Si^+^ 341.1027, found 341.1032.

### 3.4. Procedure for the Heterogeneous Synthesis of the Hydroxy-Free QC-Isotetronic Acid 12 (Scheme 3) and Protected Isotetronic Acid 2 Under Batch Conditions

Diethyl (*S*)-2-hydroxy-2-methyl-4-oxopentanedioate (**10**). To a stirred suspension of SiO_2_@Ley catalyst (725 mg, 0.45 mmol; *f* = 0.61 mmolg^−1^) in *i*-PrOH (5 mL), ethyl pyruvate **1** (500 μL, 4.50 mmol) was added dropwise. The mixture was stirred for an additional 16 h, and then filtered and concentrated to give **10** (397 mg, 76%) at least 95% pure, as judged by ^1^H NMR analysis. ^1^H NMR (400 MHz, CDCl_3_) δ = 4.32 (q, *J* = 7.2 Hz, 2H), 4.25 (q, *J* = 7.2 Hz, 2H), 3.54 (broad s, 1H), 3.38 (d, *J* = 18.2 Hz, 1H), 3.33 (d, *J* = 18.2 Hz, 1H), 1.46 (s, 3H), 1.36 (t, *J* = 7.2 Hz, 3H), 1.28 (t, *J* = 7.2 Hz, 3H). ^13^C{^1^H} NMR (126 MHz, CDCl_3_) δ = 191.9, 175.4, 160.3, 72.1, 62.8, 62.3, 48.2, 26.7, 14.1, 14.0. Spectroscopic data are in accordance with the literature [9].

The filtered SiO_2_@Ley catalyst was thoroughly washed with fresh portions of Et_2_O, dried, and then subjected to the recycle experiments (Figure 3).

Ethyl (*S*)-4-hydroxy-2-methyl-5-oxo-2,5-dihydrofuran-2-carboxylate (**12**). The above crude **10** (397 mg) was dissolved in CH_2_Cl_2_ (8 mL), and then Amberlyst 15 (1.5 g) was added in one portion. The suspension was shaken at room temperature for 3 h, and then the resin was filtered off and washed thoroughly with CH_2_Cl_2_. The combined filtrates were concentrated and dried under a high vacuum. The resulting crude material was dissolved in CH_2_Cl_2_ (12 mL), and then Amberlyst 21 (2.5 g) was added in one portion. The suspension was shaken at room temperature for 12 h, and then the resin was filtered off and washed thoroughly with CH_2_Cl_2_. Next, the resin was suspended in CH_2_Cl_2_ (8 mL), and then TFA (4 mL) was added in one portion. The resulting mixture was shaken at room temperature for 2 h. The resin was then filtered off and washed thoroughly with CH_2_Cl_2_. The combined filtrates were concentrated to give the isotetronic acid **12** in equilibrium with its dicarbonyl form (276 mg, 66% from 1; 85% ee) at least 95% pure, as determined by ^1^H NMR analysis. Selected date for **12** ^1^H NMR (400 MHz, CDCl_3_) δ = 6.25 (s, 1H), 5.20 (s, 1H), 4.23 (s, 2H), 1.73 (s, 3H), 1.29 (t, *J* = 7.1 Hz, 3H). ^13^C{^1^H} NMR (126 MHz, CDCl_3_) δ = 169.1, 168.6, 142.3, 119.0, 83.3, 62.7, 22.8, 14.0. Spectroscopic data are in accordance with the literature [9].

For the determination of **12** enantiomeric excess, the crude 10 was silylated in a parallel experiment, as described in Section 3.1, to give **2** (ee = 85%) as a clear, colorless oil. [α]_D_^20^ = –30.6 (c 1.2, CHCl_3_). HPLC analysis Chiralpak^®^ IA (*n*-Hexane:*i*-PrOH 98:2, flow rate 1 mL min^−1^, 242 nm, 20 °C) t_1_ = 4.7 min (minor), t_2_ = 5.1 min (major), 92.51:7.49 er.

### 3.5. Fabrication of Mesoreactor R

A slurry solution was prepared suspending an excess of catalyst SiO_2_@Ley in *i*-PrOH. A stainless-steel column (10 × 0.21 cm) was filled with the slurry solution under constant pressure (300 bars, 30 min, *i*-PrOH as solvent) by using an air-driven liquid pump. The microreactor void volume (V_0_) was determined using pycnometry, filling and weighting accurately the mesoreactor with two different solvents (solvent 1: *i*-PrOH; solvent 2: *n*-hexane). V_0_ was calculated according to the following formula, V_0_ = (w_1_ − w_2_)/(δ_1_ − δ_2_), where w_1_ and w_2_ are the weights of the microreactor filled, respectively, with solvents 1 and 2, and δ_1_ and δ_2_ are the solvent densities.

### 3.6. Experimental Set-Up for Flow Experiments and Continuous-Flow Synthesis of Isotetronic Acids 2, 7, and 9 (Table 4)

The system used for the flow reaction was made of one pump (Agilent 1100 micro series) connected to the reactor by a feeding channel that was used to deliver a solution of **1** (0.14 M) and either acceptor **6** (0.28 M) or **8** (2.67 M) in *i*-PrOH. The feed solution was pumped with a flow rate of 5 μL min^−1^ into R for ca. 1 h to reach the optimal steady-state regime, and then for an additional 24 h. The outlet stream was frequently analyzed for the evaluation of **1** conversion using ^1^H NMR analysis. The collected solution of the 24 h experiment was concentrated and subjected to the silylation procedure described before for the determination of the yield and enantiomeric excesses of isotetronic acids **2**, **7**, and **9**.

### 3.7. Long-Term Stability Experiment (Figure 3)

The long-term stability investigation was performed by feeding the mesoreactor R with a solution of ethyl pyruvate **1** (0.14 M) for 200 h. The outlet stream was analyzed every 12 h for the evaluation of **1** conversion using ^1^H NMR analysis. Periodically, fractions were collected to determine the enantiomeric excesses of isotetronic acid **2**.

## 4. Conclusions

In summary, we demonstrated in this study that the silica-supported version of the privileged Ley organocatalyst, namely 5-(pyrrolidin-2-yl)tetrazole, is capable of effectively promoting the self- and cross-aldol reactions of pyruvates, with the levels of conversion efficiency and enantioselectivity comparable to or even better than those displayed by the homogeneous counterpart. We also detected good recyclability of the heterogeneous catalyst in batch experiments and its remarkable long-term stability in the flow regime. To the best of our knowledge, this study represents the first example of optically active isotetronic acid synthesis using fixed-bed reactors, thus paving the way for future advances in the field of heterogeneous catalysis under flow conditions for the efficient production of biologically relevant molecules.

## Figures and Tables

**Table 1:** Self-aldol reaction of ethyl pyruvate 1 promoted by organocatalysts **3**–**5**.

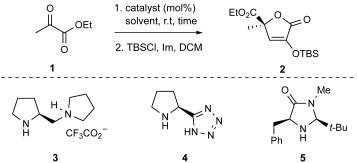					
Entry ^1^	Catalyst (mol%)	Solvent	Time	Yield (%) ^2^	ee (%) ^3^
1	**3** (30) ^4^	*i*-PrOH	16 h	59	86
2	**4** (30)	*i*-PrOH	8 h	26	61
3	**4** (20)	*i*-PrOH	12 h	31	61
4	**4** (10)	*i*-PrOH	18 h	46	62
5	**4** (5)	*i*-PrOH	5 d	35	61
6	**4** (5)	MeCN	5 d	27	49
7	**4** (5)	DMSO	5 d	32	54
8	**5**-TFA (30) ^5^	*i*-PrOH	7 d	<5	/
9	**5**-AcOH (30) ^5^	*i*-PrOH	7 d	<5	/
10	**5**-TFA (30) ^5^	MeCN	7 d	<5	/
11	**5**-AcOH (30) ^5^	MeCN	7 d	<5	/
12	**5**-TFA (30) ^5^	DMSO	7 d	<5	/
13	**5**-AcOH (30) ^5^	DMSO	7 d	<5	/

^1^ All reactions were performed with 0.90 mmol of **1** and the reported amount of catalyst in the stated solvent at room temperature. ^2^ Isolated yield of **2**. ^3^ Determined using chiral HPLC. ^4^ Ref [9]. ^5^ The ratio amine/coupled acid is 1:1.

**Table 2:** Cross-aldol reaction of ethyl pyruvate **1** and oxobutyrate **6** promoted by organocatalysts **3**–**5**.

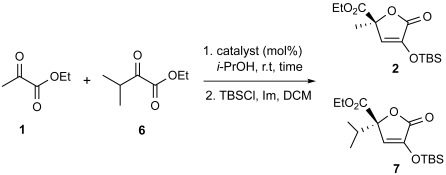					
Entry ^1^	Catalyst (mol%)	6:1	Time	Yield (%) ^2^/*ee* (%) ^3^	
				2	7
1	**3** (30)	1:1	5 d	40 (72)	10 (71)
2	**4** (30)	1:1	5 d	32 (61)	12 (98)
3	**3** (30)	10:1	7 d ^4^	15 (23)	35 (74)
4	**4** (30)	10:1	7 d ^4^	34 (55)	40 (98)
5	**4** (20)	10:1	7 d ^4^	8 (26)	29 (97)
6	**4** (30)	10:1	3 d ^4^	22 (51)	32 (95)
7	**5**-TFA ^5^ (30)	10:1	7 d ^4^	<5 (/)	<5 (/)

^1^ All reactions were performed with 0.45 mmol of **1** and the reported amount of **6** and catalyst in i-PrOH at room temperature. ^2^ Isolated yield. ^3^ Determined using chiral HPLC. ^4^ Addition of **6** completed in 36 h by a syringe pump. ^5^ The ratio amine/coupled acid is 1:1.

**Table 3:** Main features of packed-bed mesoreactor **R**.

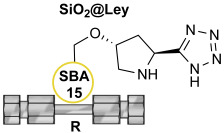					
SiO_2_@Ley (mg) ^1^	V_G_ (mL) ^2^	V_o_ [μL] ^3^	Total Porosity ^4^	Time [min] ^5^	*p* [bar] ^6^
356	346	249	0.72	25	4

^1^ Loaded catalyst: determined by calculating the difference in catalyst amount between the initial loading and the amount recovered in the residual slurry solution. ^2^ Geometric volume (V_G_) of the stainless steel column. ^3^ Void volume (V_0_) determined using pycnometry. ^4^ Total porosity ε_tot_= V_0_/V_G_. ^5^ Residence time (t_0_) calculated at 10 μLmin^−1^. ^6^ Backpressure measured at 50 μLmin^−1^ (r.t., i-PrOH).

**Table 4:** Continuous-flow production of QC-isotetronic acids **2**, **7**, and **9** ^1^.

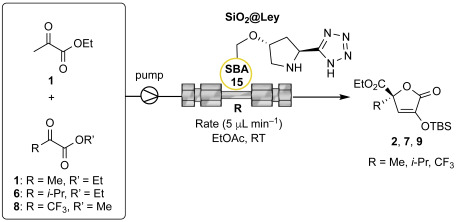							
Entry	1 (*c* [M])	Acceptor (*c* [M])	Flow Rate (μLmin^−1^) ^1^	Conversion (%) ^2^	Isotetronic Acid (%) ^3^	*ee* (%) ^4^	TOF ^5^
1	0.14	**-**	20	32	**2** (25)	86	1.5
2	0.14	**-**	10	78	**2** (70)	86	3.7
3	0.14		5	>95	**2** (91)	86	4.8
4	0.14	**6** (2.67)	5	>95	**7** (76)	98	4.8
5	0.14	**8** (0.28)	5	>95	**9** (82)	75	4.8

^1^ See the Materials and Methods section for a description of the flow apparatus. ^2^ Instant conversion of **1** in the steady-state regime, as determined using ^1^H NMR analysis of the crude aldol mixture. ^3^ Isolated yield after protection by TBS. ^4^ Determined using chiral HPLC. ^5^ Turnover frequency (TOF) is measured in mmol_prod_ d^−1^ mmol_cat_^−1^ and is referred to as the key aldol step.

## Data Availability

Data are contained within the article.

## Supplementary Materials

The following supporting information can be downloaded at https://www.mdpi.com/article/10.3390/molecules30020296/s1: Screening of substituted α-keto esters as suitable acceptors, NMR spectra, and HPLC chromatograms.
