# Supplementary material for: Organocatalytic Packed-Bed Reactors for the Enantioselective Flow Synthesis of Quaternary Isotetronic Acids by Direct Aldol Reactions of Pyruvates
Source: Molecules. 2025 Jan 13;30(2):296. doi: 10.3390/molecules30020296 (PMC11767881; doi:10.3390/molecules30020296)
Supplement: Supplementary file 1 [file molecules-30-00296-s001.zip › molecules-3416659-supplementary.pdf]

Supporting materials

Organocatalytic Packed-Bed Reactors for the Enantioselective Flow Synthesis of Quaternary Isotetronic Acids by the Direct Aldol Reactions of Pyruvates

Lorenzo Poletti,[a] Carmela De Risi,[b] Daniele Ragno,[b] Graziano Di Carmine,[a] Riccardo Tassoni,[c] Alessandro Massi,[b,*] and Paolo Dambruoso[c,*]

[a] Department of Environmental and Prevention Sciences, University of Ferrara, Via L. Borsari, 46, 44121 Ferrara (Italy)

[b] Department of Chemical, Pharmaceutical and Agricultural Sciences, University of Ferrara, Via L. Borsari, 46 – 44121 Ferrara (Italy)

[c] Institute for Organic Synthesis and Photoreactivity of the Italian National Research Council, Area della Ricerca di Bologna, Via P. Gobetti, 101 – 40129 – Bologna (Italy)

Table of contents

1. Screening of substituted α-keto esters as suitable acceptors S2
2. NMR spectra S4
3. HPLC chromatograms S10

Screening of substituted α-keto esters as suitable acceptors for cross-aldol reaction with ethyl pyruvate

The potential substrates **2**, **13**, and **14** were considered for this study. The unbranched bromo- and benzyl-substituted alkyl glyoxylates **13** and **14** acted as acceptor and donor partners in the homoaldol reaction manifold, as work-up and elaboration of the reaction mixtures afforded the corresponding *O*-silyl isotetronic acids **15** and **16**. By contrast, the branched *iso*-propyl derivative **2** remained unaltered under amine catalysis.

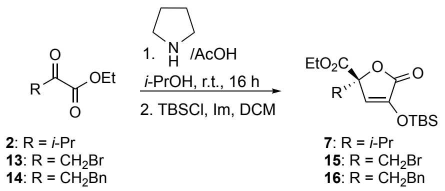

(R/S)-3-Bromo-2-(bromomethyl)-4-(tert-butyldimethyl-silanyloxy)-5-oxo-2,5-dihydrofuran-2-carboxylic Acid Ethyl Ester **(15)**. To a stirred solution of pyrrolidine (23 μL, 0.27 mmol) in *i*-PrOH (1 mL) acetic acid (15 μL, 0.27 mmol) was added in one portion. The solution was stirred at room temperature for 15 min then ethyl bromopyruvate **13** (113 μL, 0.90 mmol) was added dropwise. The mixture was stirred at room temperature for an additional 16h then concentrated. The resulting residue was dissolved in anhydrous CH_2Cl_2 (4 mL) then imidazole (184 mg, 2.70 mmol) and *tert*-butyldimethylsilyl chloride (271 mg, 1.80 mmol) were added sequentially. The mixture was stirred at room temperature for 18 h, then concentrated and eluted from a column of silica gel with 15:1 cyclohexane-AcOEt to give **15** (54 mg, 26%) as a racemic compound. ^{1}H NMR (400 MHz, $CDCl_3$) δ = 4.36-4.26 (m, 2 H, OCH_2CH_3), 4.02 and 3.98 (2 d, 2 H, *J* = 11.5 Hz, CH_2Br), 1.32 (t, 3 H, *J* = 7.0 Hz, OCH_2CH_3), 1.02 (s, 9 H, *t*-Bu), 0.35 and 0.32 (2 s, 6 H, $(CH_3)_2Si$). $^{13}C\{^1H\}$ NMR (101 MHz, $CDCl_3$): δ = 165.5, 165.1, 144.0, 117.2, 84.0, 63.8, 32.3, 25.6, 18.4, 14.2, -3.9, -4.0.

(R/S)-3-Benzyl-4-(tert-butyldimethyl-silanyloxy)-5-oxo-2-phenethyl-2,5-dihydrofuran-2-carboxylic Acid Ethyl Ester **(13)**. To a stirred solution of pyrrolidine (23 μL, 0.27 mmol) in *i*-PrOH (1 mL) acetic acid (15 μL, 0.27 mmol) was added in one portion. The solution was stirred at room temperature for 15 min then 2-oxo-4-phenylbutyrate **10** (170 μL, 0.90 mmol) was added dropwise. The mixture was stirred at room temperature for an additional 16h then concentrated. The resulting residue was dissolved in anhydrous CH_2Cl_2 (4 mL) then imidazole (184 mg, 2.70 mmol) and *tert*-butyldimethylsilyl chloride (271 mg, 1.80 mmol) were added sequentially. The mixture was stirred

at room temperature for 18 h, then concentrated and eluted from a column of silica gel with 15:1 cyclohexane-AcOEt to give **16** (68 mg, 40%) as a racemic compound. ^{1}H NMR (400 MHz, $CDCl_3$) δ =7.40-7.10 and 7.00-6.80 (2 m, 10 H, 2 Ph), 3.96-3.82 (m, 2 H, OCH_2CH_3), 3.72 and 3.64 (2 d, 2 H, J = 12.5 Hz, $PhCH_2C$=C), 2.60-2.20 and 2.15-1.90 (2 m, 4H, 2 CH_2), 1.14 (t, 3 H, J = 7.0 Hz, OCH_2CH_3), 0.98 (s, 9 H, *t*-Bu), 0.36 and 0.35 (2 s, 6 H, $(CH_3)_2Si$). $^{13}C\{^{1}H\}$ NMR (101 MHz, $CDCl_3$): δ = 168.6, 168.5, 140.7, 140.4, 137.5, 136.6, 129.1-128.6 (8C), 127.2, 126.4, 86.3, 62.5, 36.2, 30.3, 29.3, 25.9, 18.5, 14.1, -3.8, -3.9.

NMR Spectra

^{1}H-NMR (400 MHz), ^{13}C{^{1}H}-NMR (101 MHz) of *Ethyl (S)-4-((tert-butyldimethylsilyl)oxy)-2-methyl-5-oxo-2,5-dihydrofuran-2-carboxylate* (2)

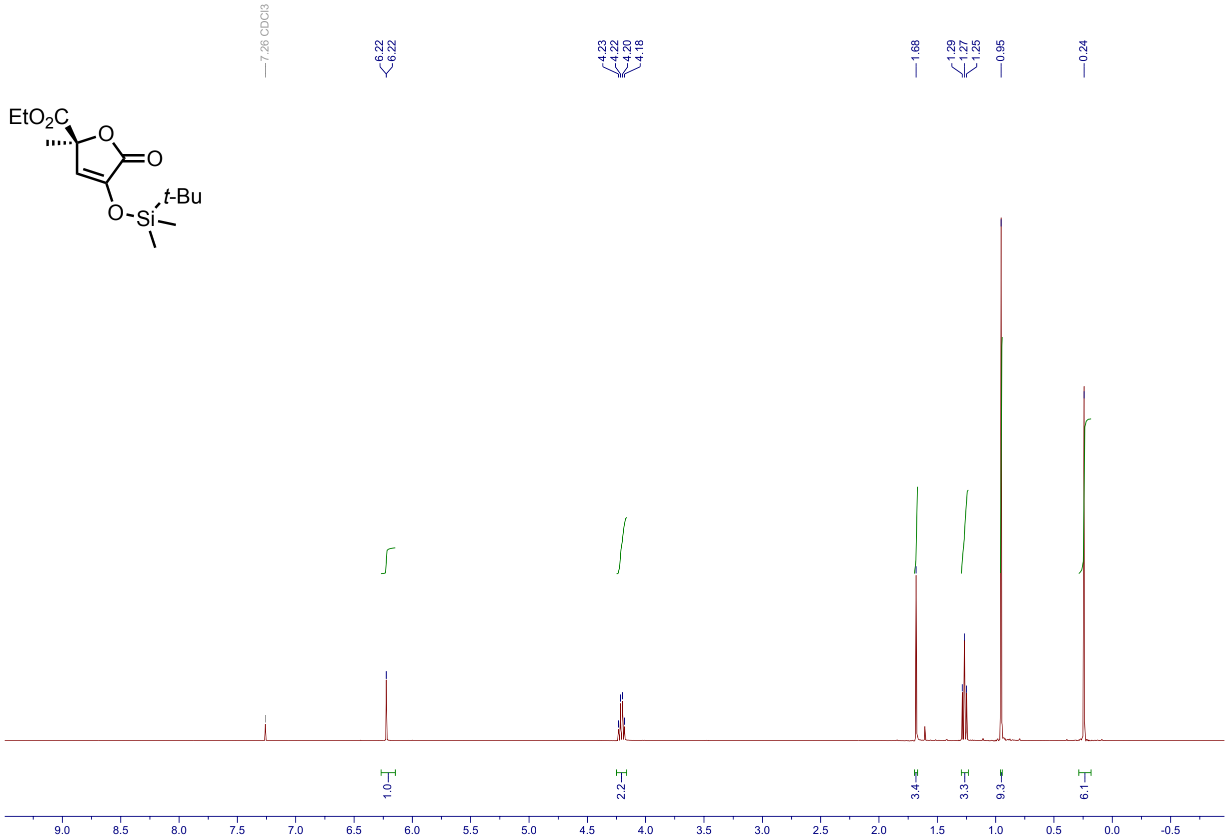

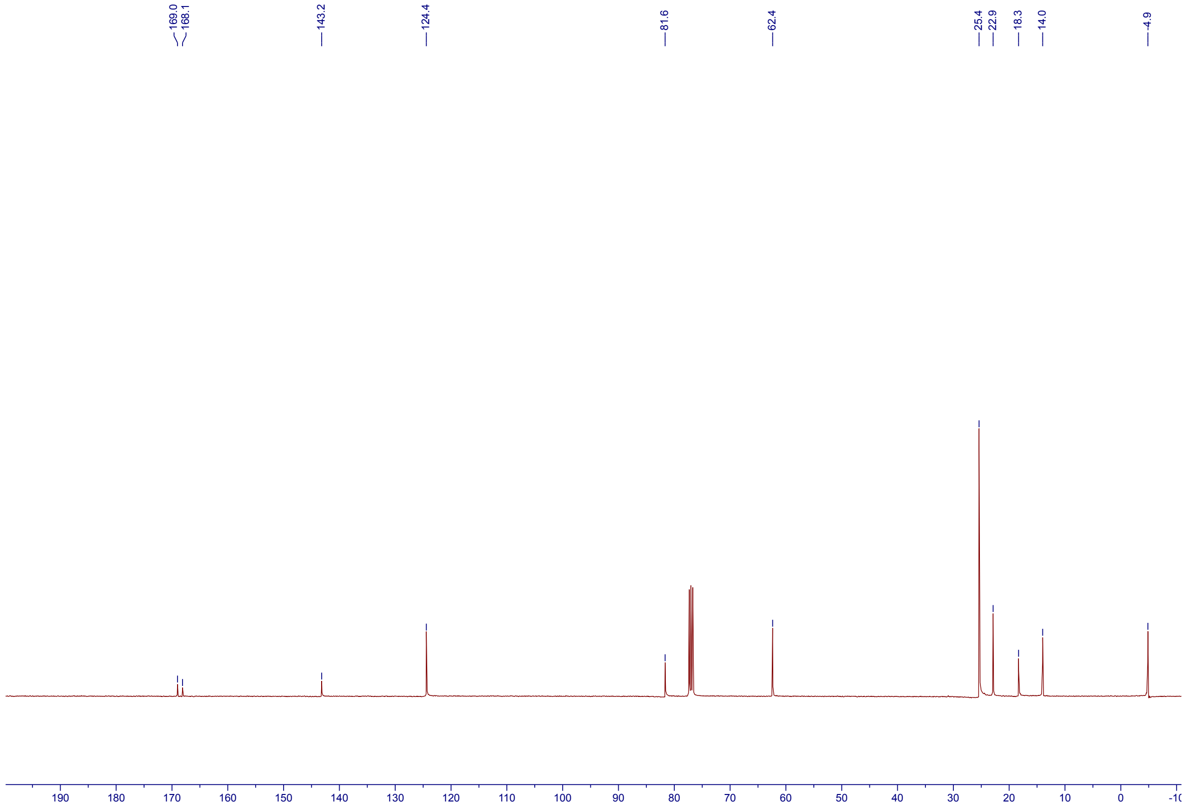

^{1}H-NMR (400 MHz), ^{13}C{^{1}H}-NMR (101 MHz) of *Ethyl (S)-4-((tert-butyldimethylsilyl)oxy)-2-isopropyl-5-oxo-2,5-dihydrofuran-2-carboxylate* (7)

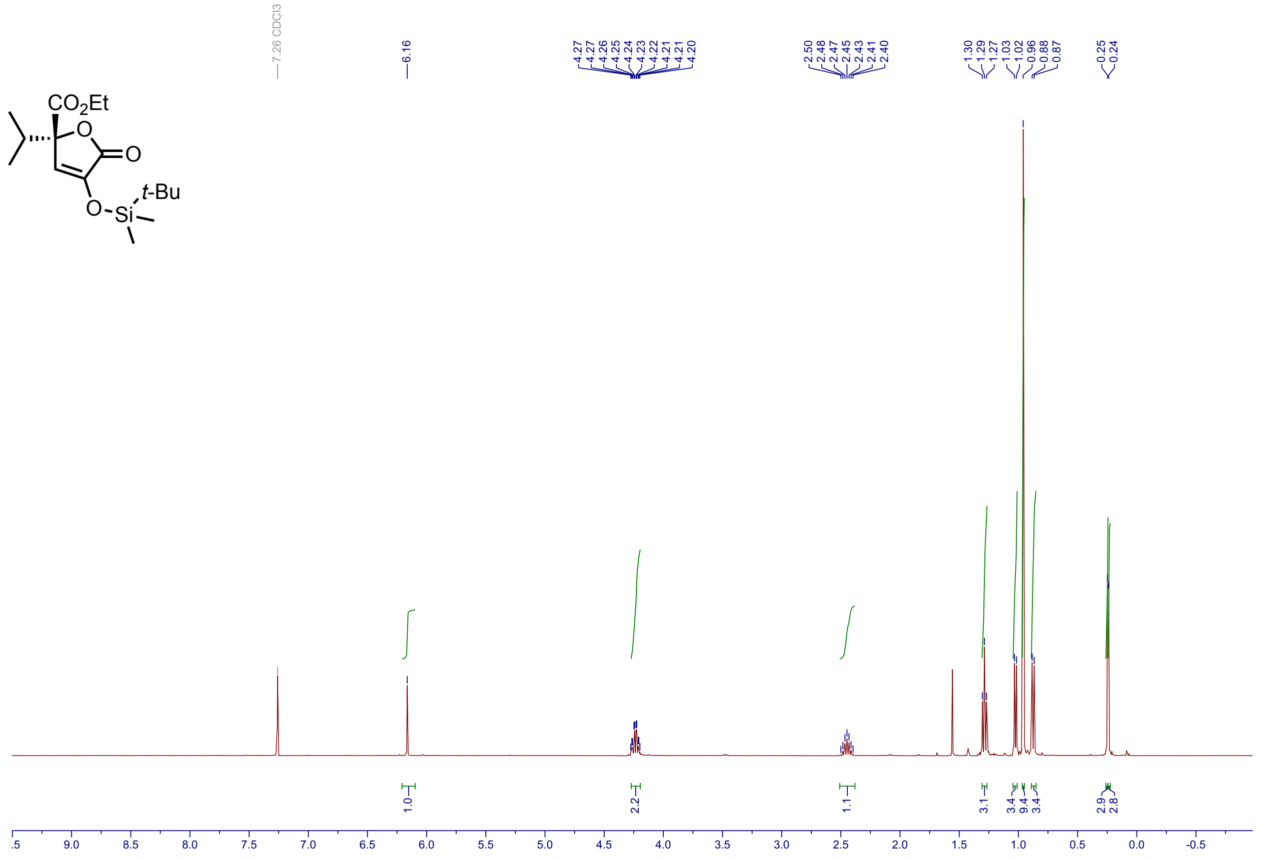

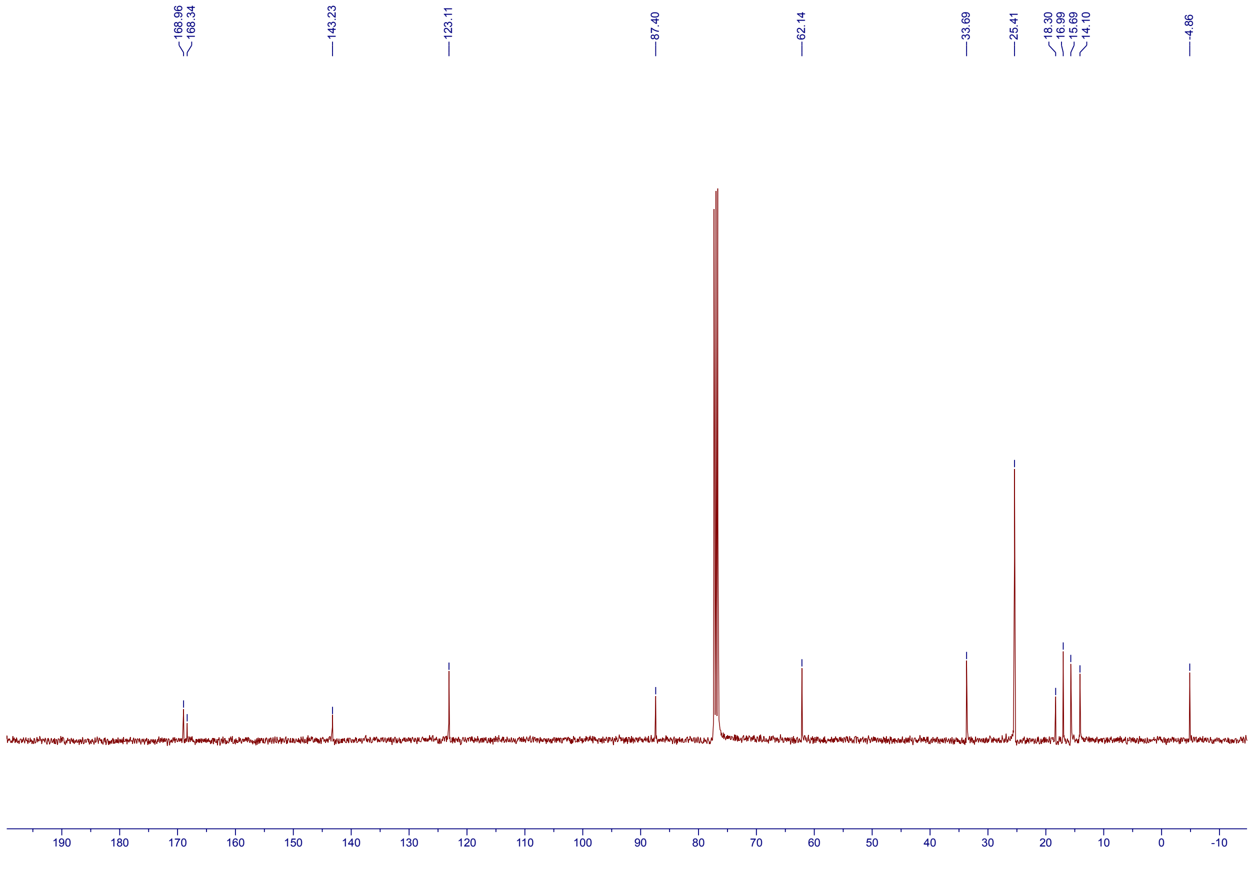

^{1}H-NMR (400 MHz), ^{13}C{^{1}H}-NMR (126 MHz), ^{19}F (471 MHz) of *Methyl (S)-4-((tert-butyldimethylsilyl)oxy)-5-oxo-2-(trifluoromethyl)-2,5-dihydrofuran-2-carboxylate* (9)

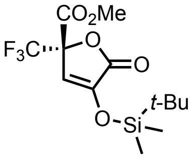

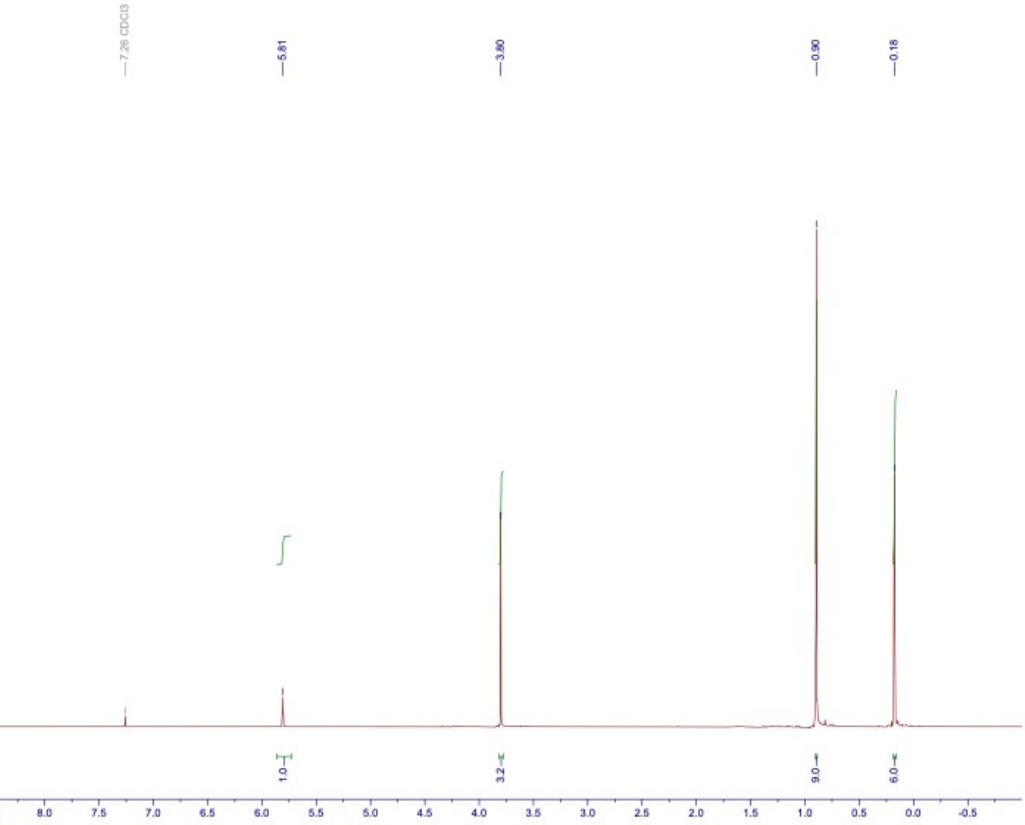

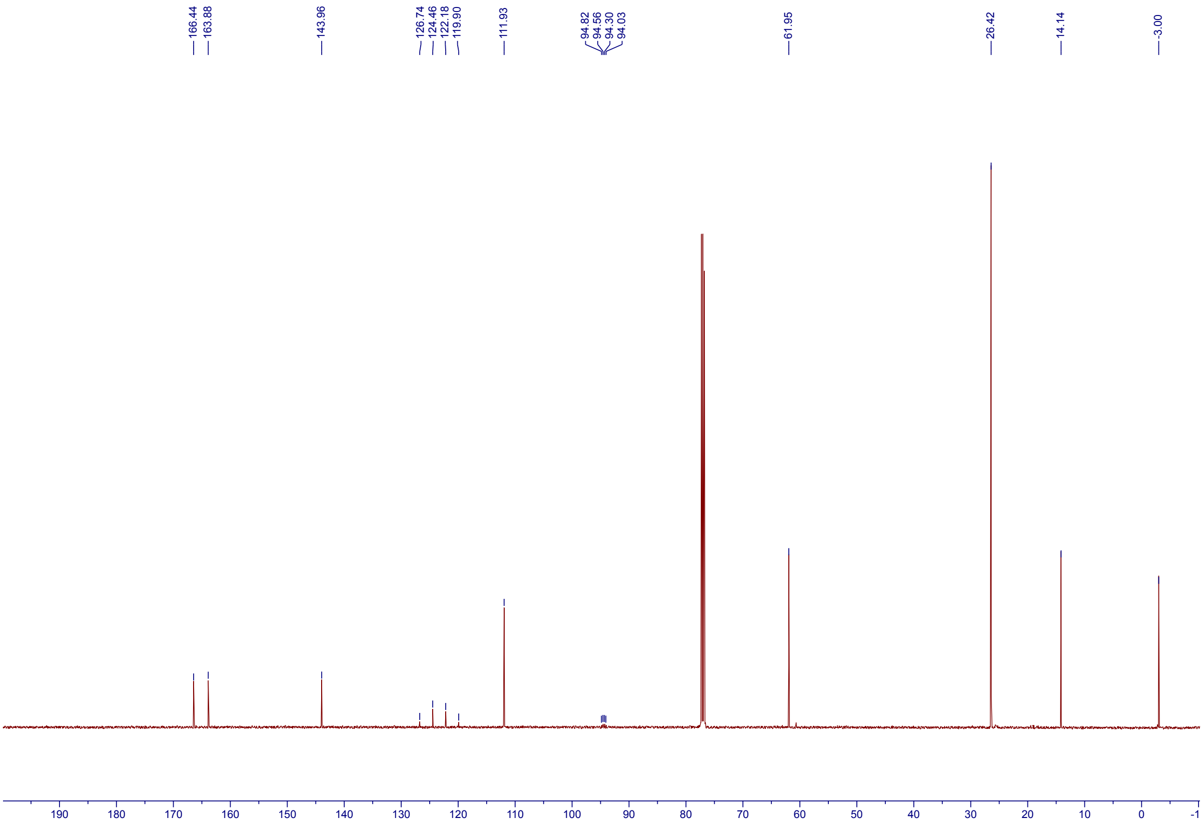

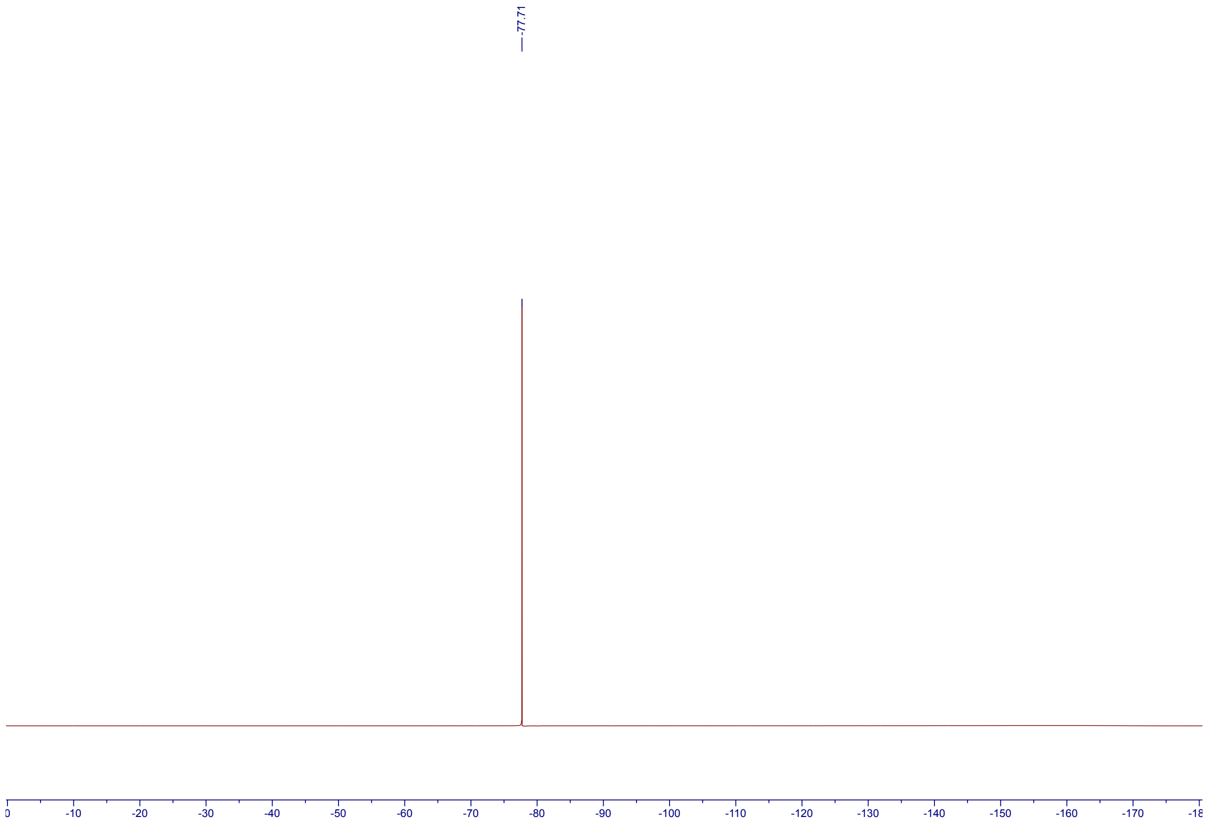
-77.71
0
-10
-20
-30
-40
-50
-60
-70
-80
-90
-100
-110
-120
-130
-140
-150
-160
-170
-18

^{1}H-NMR (400 MHz), ^{13}C{^{1}H}-NMR (126 MHz) of (*S*)-2-Hydroxy-2-methyl-4-oxoglutarate (10)

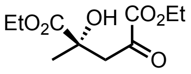

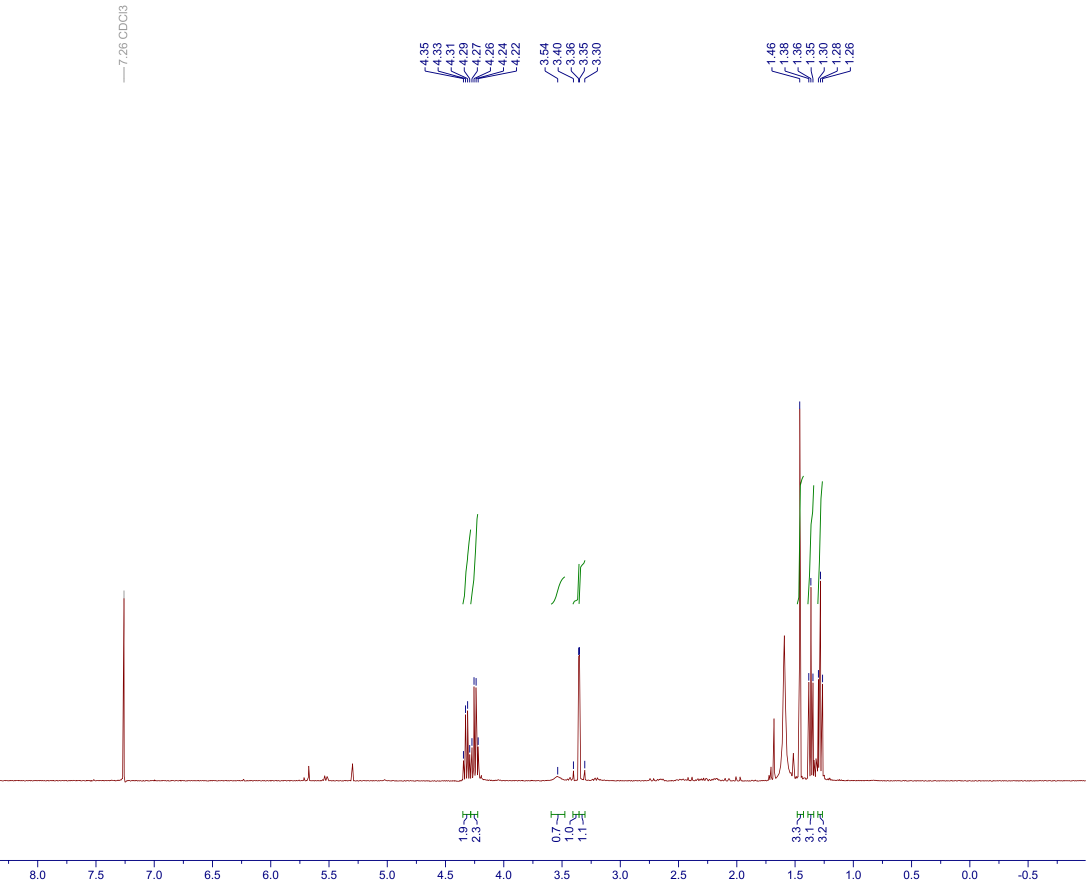

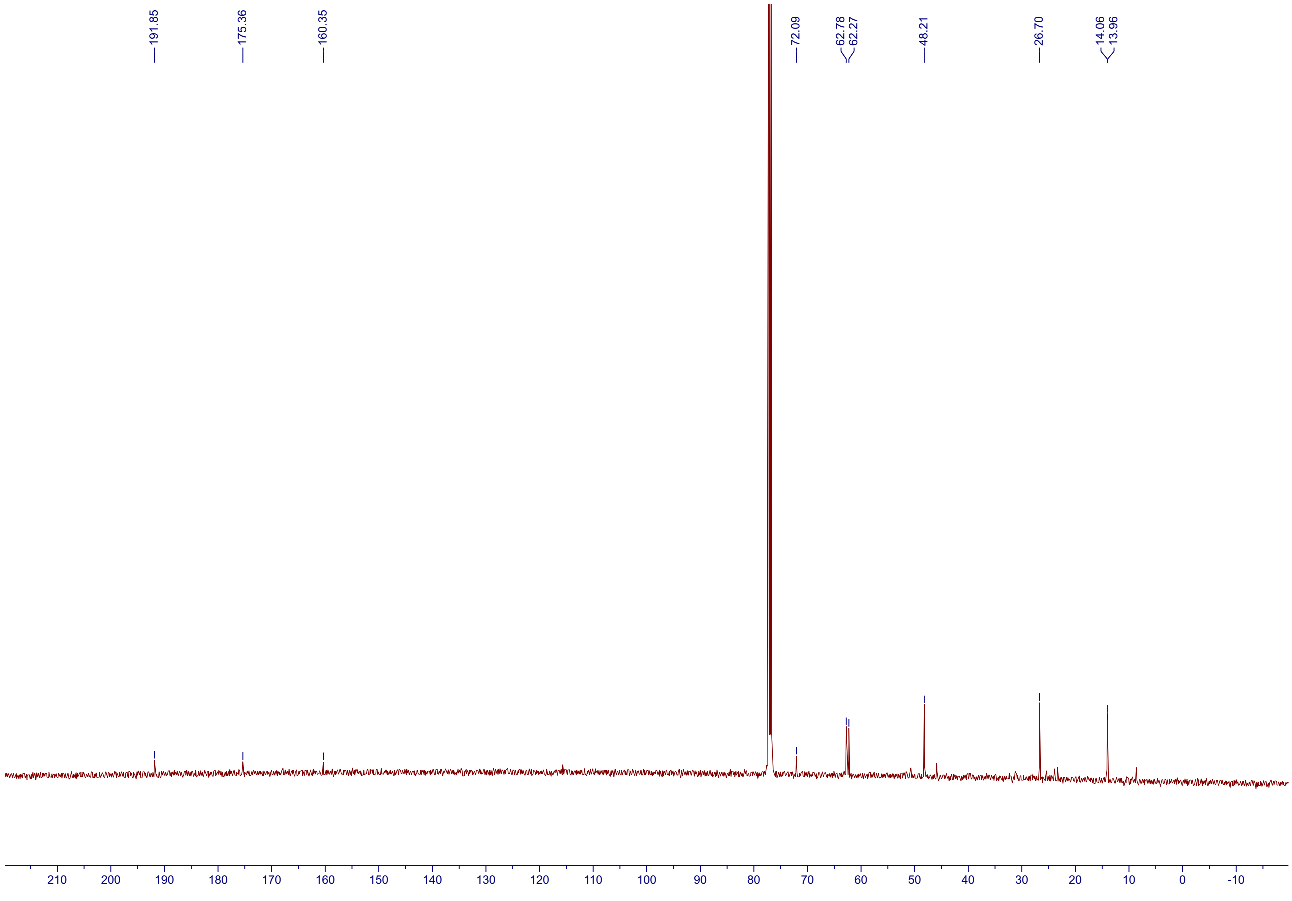

^{1}H-NMR (400 MHz), ^{13}C{^{1}H}-NMR (126 MHz) of *Ethyl (S)-4-hydroxy-2-methyl-5-oxo-2,5-dihydrofuran-2-carboxylate* (12) in equilibrium with its dicarbonyl form.

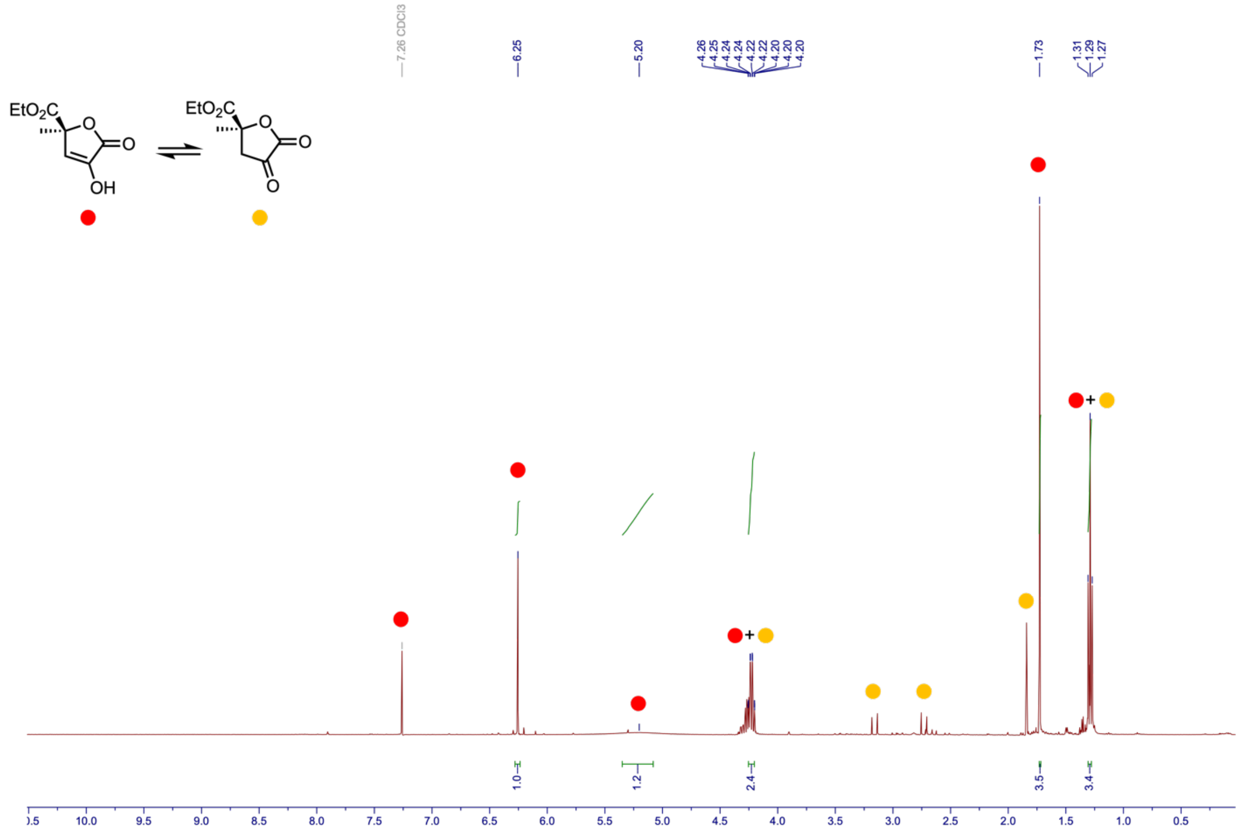

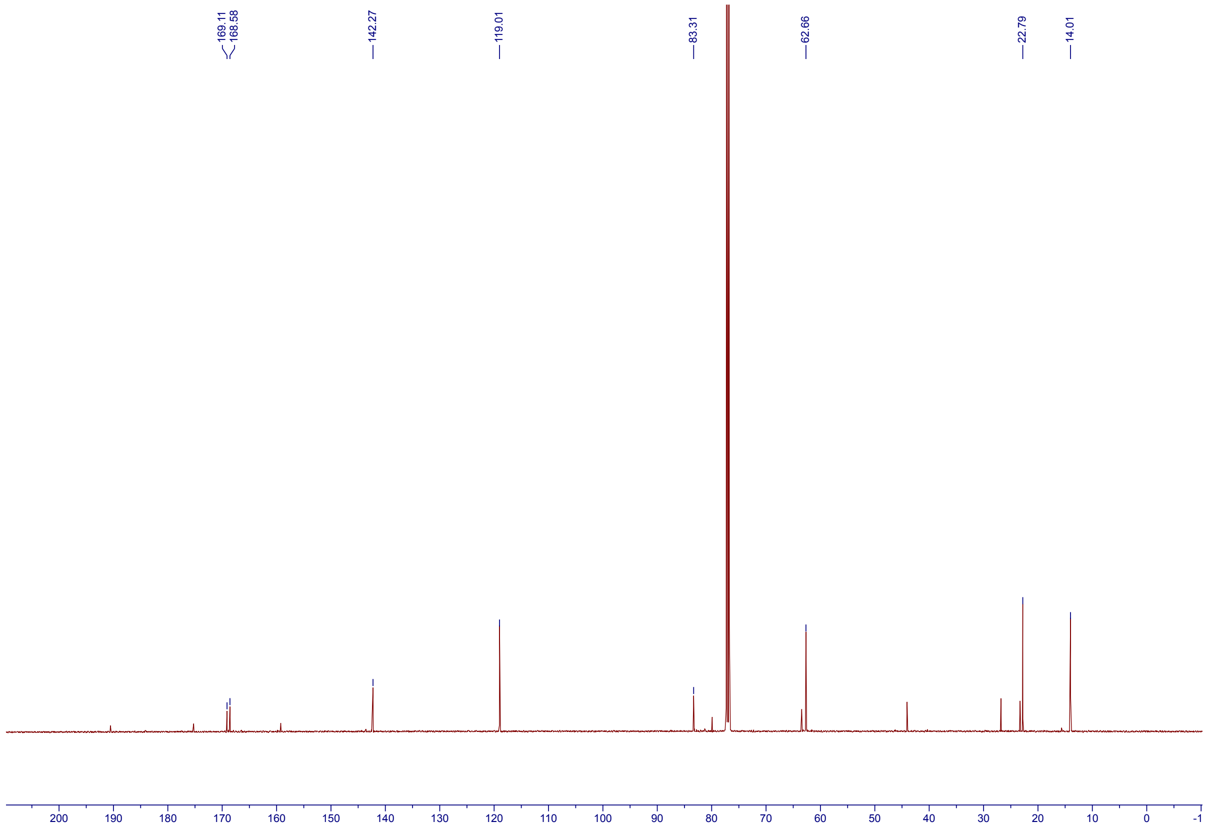

HPLC chromatograms

Ethyl (S)-4-((tert-butyldimethylsilyl)oxy)-2-methyl-5-oxo-2,5-dihydrofuran-2-carboxylate **(2)**.

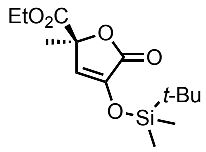

Chiral HPLC analysis Chiralpak® IA (Hexane:i-PrOH 98:2, flow rate 1 mL min^{-1}, 242 nm, 20 °C) 7.49:92.51 er.

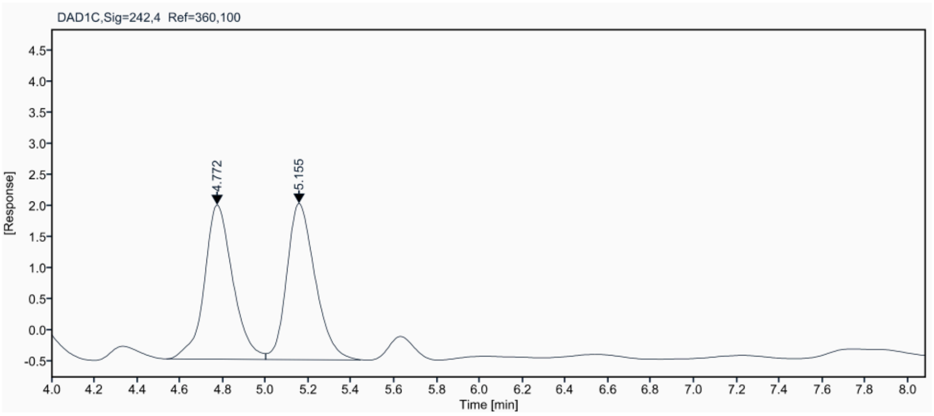

RT [min]	Type	Width [min]	Area	Height	Area%	Name
4.772	BV	0.4638	23.0163	2.4922	49.8128	
5.155	MF m	0.4428	23.1893	2.5222	50.1872	

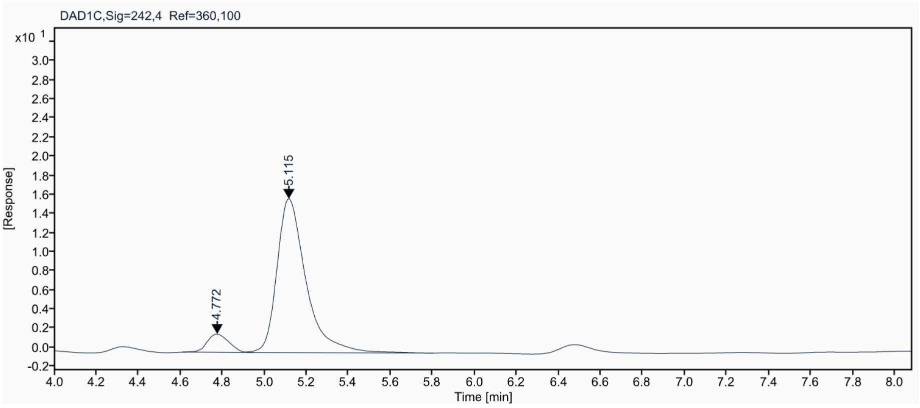

RT [min]	Type	Width [min]	Area	Height	Area%	Name
4.772	MM m	0.3401	12.5013	1.8514	7.4896	
5.115	MM m	0.8564	154.4145	16.1136	92.5104	

Ethyl (*S*)-4-((tert-butyldimethylsilyl)oxy)-2-isopropyl-5-oxo-2,5-dihydrofuran-2-carboxylate (**7**)

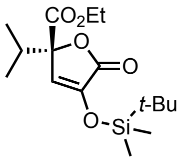

Chiral HPLC analysis Chiralpak® IA (Hexane:i-PrOH 97:3, flow rate 1 mL min^{-1}, 242 nm, 20 °C) 0.92:99.08 er.

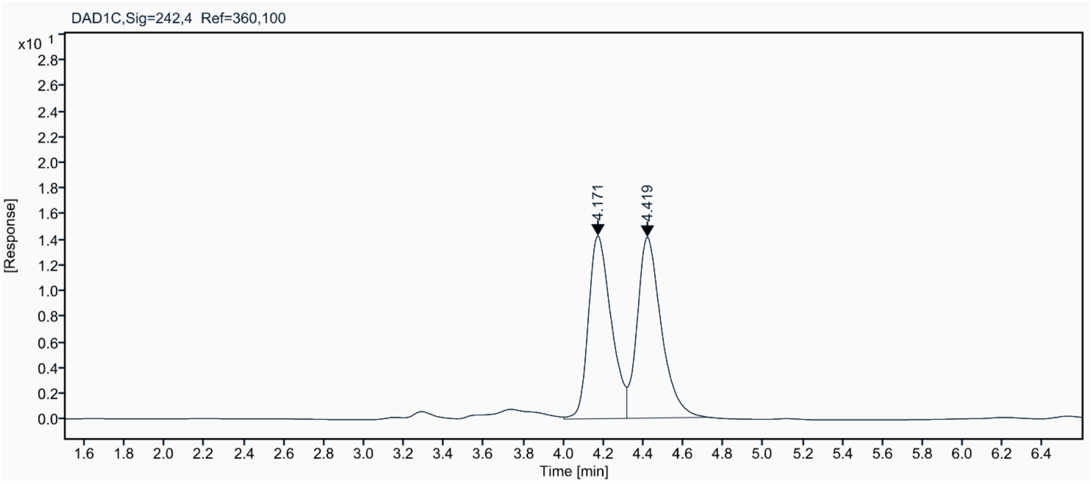

RT [min]	Type	Width [min]	Area	Height	Area%
4.171	VM m	0.3161	113.5328	14.2651	48.8632
4.419	MM m	0.4182	118.8152	14.1344	51.1368

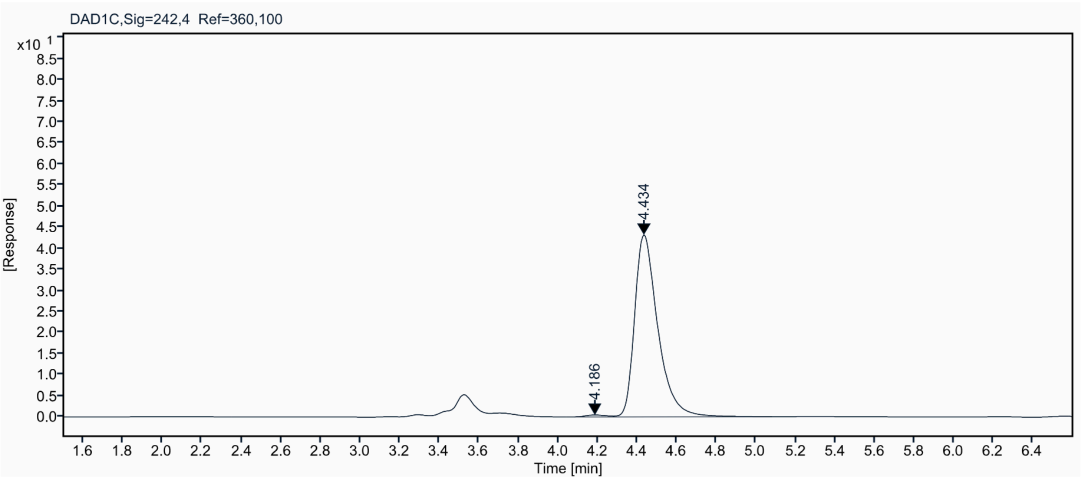

RT [min]	Type	Width [min]	Area	Height	Area%
4.186	MM m	0.1984	3.2582	0.4561	0.9199
4.434	VB	0.7984	350.9295	43.2148	99.0801

Methyl (*S*)-4-((tert-butyldimethylsilyl)oxy)-5-oxo-2-(trifluoromethyl)-2,5-dihydrofuran-2-carboxylate (**9**).

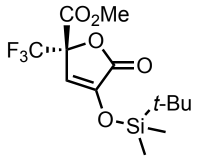

Chiral HPLC analysis Chiralpak® IA (Hexane:i-PrOH 98:2, flow rate 1 mL min^{-1}, 242 nm, 20 °C) 87.51:12.49 er.

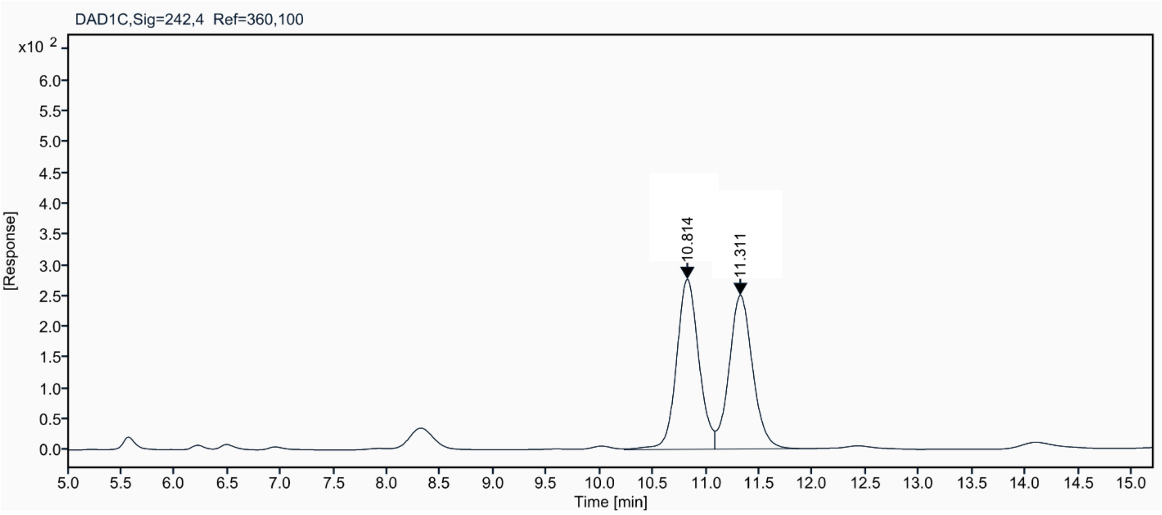

RT [min]	Type	Width [min]	Area	Height	Area%
10.814	VM m	0.8519	4015.8718	276.0657	51.1862
11.311	MM m	0.7927	3829.7476	249.7712	48.8138

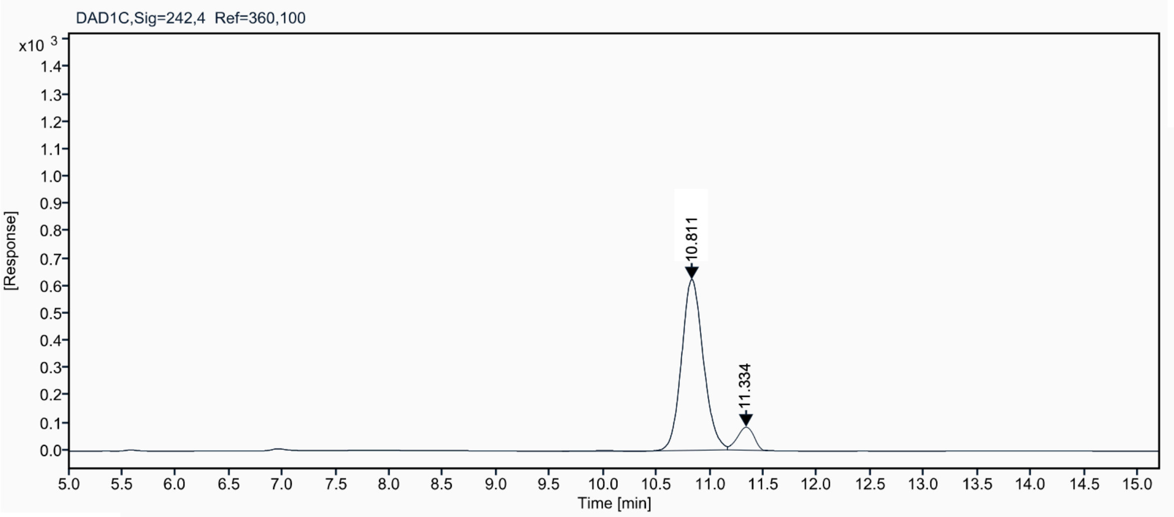

RT [min]	Type	Width [min]	Area	Height	Area%
10.811	MM m	0.7329	8836.1425	621.2412	87.5124
11.334	MM m	1.0222	1260.8751	99.4315	12.4876
